# Edge-emitting polariton laser and amplifier based on a ZnO waveguide

**DOI:** 10.1038/s41377-018-0084-z

**Published:** 2018-10-31

**Authors:** O. Jamadi, F. Reveret, P. Disseix, F. Medard, J. Leymarie, A. Moreau, D. Solnyshkov, C. Deparis, M. Leroux, E. Cambril, S. Bouchoule, J. Zuniga-Perez, G. Malpuech

**Affiliations:** 1 Institut Pascal, PHOTON-N2, Université Clermont Auvergne, CNRS, SIGMA Clermont, F-63000 Clermont-Ferrand, France; 2 UCA, CRHEA-CNRS, Valbonne, F-06560 France; 3 Centre Nanosciences et Nanotechnologies (C2N), CNRS, University Paris-Saclay, Marcoussis, F-91460 France

## Abstract

We demonstrate edge-emitting exciton-polariton (polariton) laser operation from 5 to 300 K and polariton amplifiers based on polariton modes within ZnO waveguides. The guided mode dispersion below and above the lasing threshold is directly measured using gratings placed on top of the sample, fully demonstrating the polaritonic nature of the lasing modes. The threshold is found to be smaller than that expected for radiative polaritons in planar ZnO microcavities below 150 K and comparable above. These results open up broad perspectives for guided polaritonics by enabling easier and more straightforward implementation of polariton integrated circuits that exploit fast propagating polaritons, and, possibly, topological protection.

## Edge-Emitting Polariton lasers: A step closer to two-dimensional all-optical devices

A type of laser that can propagate along tiny, two-dimensional horizontal cavities in semiconductor materials at room temperature could be used in novel all-optical devices. Polaritons are quasi-particles created from the resonant coupling of light (photons) and matter (electron-hole pairs, or ‘excitons’) in tiny enclosed spaces, such as the microcavities fabricated from semiconductor materials. Polariton lasers could enable researchers to build energy-efficient, compact all-optical devices. Guillaume Malpuech at the Institut Pascal in Clermont-Ferrand, France, and co-workers have designed a new type of polariton laser and amplifier operating at room temperature. Their design does not require the use of a microcavity but is based on the use of the polariton guided modes of a ZnO layer. This horizontal, guided geometry is appealing because it is easy to fabricate and to integrate in two-dimensional optical circuits.

## Introduction

Exciton-polaritons (polaritons) are quasi-particles that result from the coupling between a light mode and an excitonic resonance. Polaritons were theoretically introduced by Hopfield^1^ and Agranovich^2^ at the end of the 50′s to describe light propagation in bulk semiconductors. In 1992, the achievement of strong light-matter coupling between the photonic radiative modes of a planar microcavity and the excitonic resonances of embedded quantum wells^3^ opened up the era of two-dimensional cavity polaritons, which have since been extensively studied. From the fundamental side, polaritons represent a direct implementation of a spinor quantum fluid of light^4,5^, with unique access to the time-dependent wavefunction in real and reciprocal space via optical measurements, which enables study of Bose-Einstein condensation in open systems^6-8^, of the superfluidity of light^9,10^, and of various types of topological defects^11,12^. In-plane potentials have been commonly realized for a decade. These potentials enable one to implement coupled 0D polariton modes, building artificial molecules^13^ or lattices^14-17^, which opens up new perspectives for emulating different physical systems, such as topological insulators^18^, topological polariton lasers^19,20^, or the classical XY model^21^. From the applied side, polaritons have the critical advantage of having a high nonlinear response, low-threshold operation, and potentially high scalability^10^ and are useful for realizing low-consumption, compact, all-optical devices, which could replace electronics for some tasks.

The most paradigmatic polariton device is the so-called polariton laser^22^, based on polariton condensation, which does not require electron-hole gain and can therefore exhibit a very low threshold. In GaAs-based samples, at 5K, a wide variety of devices, such as switches and optical transistors, have been demonstrated (for a review see ref. ^10^). Most of these devices are based on the creation of a polariton flow based on slow radiative modes propagating at 1–2% of the speed of light, which is only possible at low temperature in very high Q samples (typically *Q*~10^5^). Room-temperature polaritonics requires the use of microcavities based on large-bandgap semiconductors (GaN, ZnO) or various organic materials, where polariton lasing has also been demonstrated^23-28^. However, their Q-factors remain limited to a few thousand, which makes it difficult to implement room-temperature polariton-based switches that exploit concepts developed in GaAs-based samples.

An alternative geometry for polaritonics is one where either Bloch surface waves^29-31^, or guided modes are confined by total internal reflection in a layer^32-38^, strongly coupled to excitonic resonances. The geometry is very appealing because of its simple technological realization, easier electrical injection, and the possibility it opens up to design integrated polaritonic circuits with very limited radiative losses. Guided polaritons have been observed and studied in GaAs^33,34^, organic materials^30,35^, GaN^36^, and transitional metal dichalcogenides^37^ (TMD), with recent reports of nonlinear polariton–polariton interaction^31,38^. This horizontal geometry is very favorable for straightforward implementation of polariton integrated circuits with the use of topological protection, such as in the recently reported “topological lasers”^39,40^. However, even if theoretically predicted^32^, “horizontal” edge-emitting polariton lasing in such fast-propagating polariton modes has never been observed.

In this work, we realize one of the crucial milestones of polaritonics by reporting lasing in ZnO-based waveguides from 5 to 300 K and the amplification of guided polariton modes under optical pumping. The mode dispersion below and above the lasing threshold is directly measured using gratings placed on the top of the sample. These dispersions clearly demonstrate the polaritonic nature of the lasing modes.

## Results

**Fig. 1:**
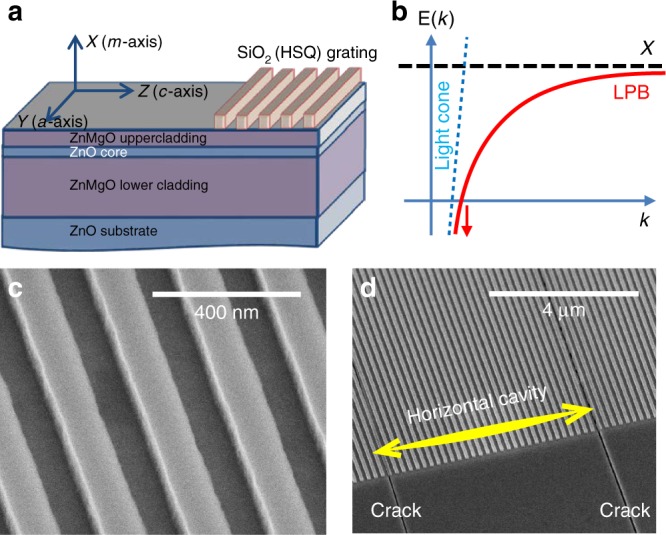
Description of sample W1. **a** Scheme for the sample. The guided mode propagates along the *Z*-axis in the ZnO core. **b** Sketch of the lower branch (LPB) of an exciton-polariton guided mode. The arrows symbolize the absence of a finite energy minimum. **c** Scanning electron microscope (SEM) image of the SiO_2_ grating deposited on top of the waveguide. **d** SEM image of the sample showing two typical horizontal cracks here separated by 18 µm, nearly parallel to the grating

We use two ZnO–ZnMgO waveguides grown by molecular beam epitaxy onto m-plane bulk ZnO substrates. The thicknesses of the active ZnO layers are 50 nm (W1 sample) and 130 nm (W2 sample), respectively. A sketch of the sample W1 is shown in Fig. 1a, whereas a sketch of the guided exciton-polariton dispersion (lower branch) is shown in Fig. 1b. At low energies, the polariton dispersion is photon-like: linear in wave vector and showing no energy minimum, which is a key difference with respect to 2D cavity polaritons. W1 is covered by sets of SiO_2_ gratings (Fig. 1c), perpendicular to the ZnO *c*-axis, with period Λ. The shift of the propagation constant by 2*π*/*Λ* allows direct access to the polariton guided mode dispersion. The sample W2 is a half-microcavity without any grating. The sample geometries together with fabrication details are presented in Supplementary material and in ref. ^41^. The common aspect of both samples is the presence of regular horizontal cracks, as one can see in the SEM image (Fig. 1d). These cracks appear because of the mismatch of the lattice parameter and the thermal expansion coefficient between ZnO and ZnMgO. They occur perpendicular to the *c*-axis of the crystal and are typically separated by 5–40 µm. By reflecting light they induce a substantial confinement leading to the formation of a genuine horizontal cavity for the guided modes. Their presence plays a crucial role in the success of our observations compared with the previous experimental studies of polariton waveguides.

**Fig. 2:**
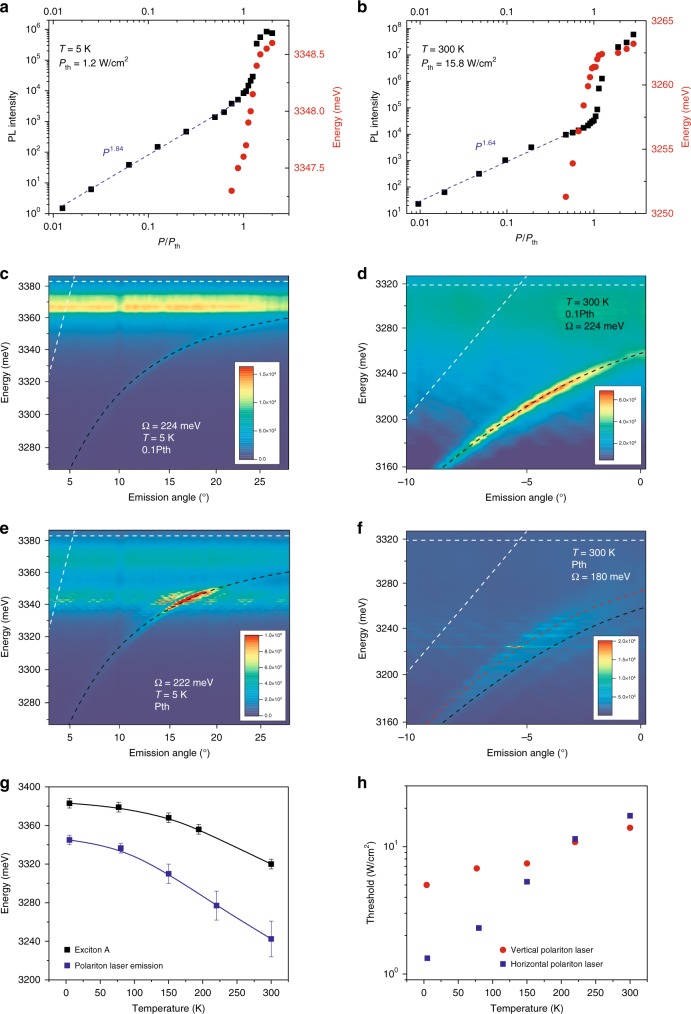
Emission of W1 at 5 and 300 K. The pump spot covers the entire horizontal cavity length. **a** 5 K. Power dependence of the emission (black squares) and energy at the wave vector of the most intense lasing peaks. **b** Same as **a**, but at 300 K. **c**–**f** Energy of emission vs. angle (dispersion). The white dashed lines are the expected bare TE0 photon and exciton modes. The black dashed lines are the strongly coupled polariton modes with the *Ω*_R_ indicated in each panel. The intensities are given in arbitrary units. **c** 5 K and 0.1 *P*_th_, **d** 300 K and 0.1 *P*_th_, **e** 5 K and *P*_th_, **f** 300 K and *P*_th_. The red dashed lines in **f** is the polariton dispersion with *Ω*_R_ = 224 meV. **g** Experimental average exciton energies plotted together with the average horizontal polariton laser emission energies vs. temperature. The solid black line corresponds to Viña’s law and the blue line is a guide for the eye. **h** Polariton lasing threshold vs. temperature for a horizontal polariton laser in W1 (blue squares) and for a vertical cavity polariton laser studied in ref. ^48^ (red stars)

The micro-photoluminescence of the samples is studied by quasi-cw excitation using 400-ps-long pulses (Methods section), much longer than all relevant time scales in the system, which enables the establishment of a steady state regime. The power dependencies of the emission of W1 at 5 and 300 K are shown in Fig. 2a, b, demonstrating a very clear nonlinear threshold at *P*_th_. The horizontal cavity sizes are 23 µm and 27 µm at 5 K and 300 K, respectively. They are entirely covered by the pump spot and we do not use spatial selection of the emission, which originates from both the grating and the cracks. The guided polariton mode dispersions at 5 and 300 K, below and above threshold, are shown in Figs. 2c–f, respectively. The optimal grating periods are *Λ* = 190 nm at 5 K and *Λ* = 180 nm at 300 K. All measured dispersions clearly deviate from the bare photonic mode (shown by the dashed line together with the bare A-exciton energy). At 5 K, the main emission below threshold arises from donor-bound excitons (D^0^X) located ~15 meV below the A-exciton energy. The emission from the cracks appears as a weak flat emission line, which can be removed by spatially selecting the emission from the grating only (Supplementary Fig. S2). The measured polariton dispersion is well reproduced by a two-oscillator model where both A and B excitonic resonances are modeled as a single oscillator placed at the A-exciton energy. We take a linear photon dispersion with a slope fixed by that of the measured dispersions at ~3.1 eV. The extracted Rabi splitting is *Ω*_R_ = 224 meV at low pumping for both temperatures. At *P*_th_, the dispersion remains essentially unchanged at 5 K (*Ω*_R_ = 222 meV). Polariton lasing takes place in modes having an exciton fraction of ~90%. A movie with emission images vs. angle and energy over a wide pumping range (0.1–20 *P*_th_) is presented in the Supplementary material. An increase in the pumping intensity induces a blueshift for the modes, as shown in Fig. 2a, b and Supplementary Fig. S3, due to screening of the exciton oscillator strength. This screening effect is more significant at 300 K because of the larger pumping threshold value, with the fit giving *Ω*_R_ = 180 meV, which is 20% weaker than at low pumping (Fig. 2f). Polariton lasing occurs in more photon-like states than at 5 K, as theoretically expected^32^, but still with a substantial 50–60% excitonic fraction. The velocity of these modes is ~40–50% of that of the bare photonic mode of the waveguide, ~60 µm/ps. In general, an increase of the pumping density leads to a blueshift of the dispersion due to the screening effect as well as to a faster polariton relaxation along this dispersion. In 2D cavities, polaritons cannot relax energetically below their ground state, and the blueshift of the dispersion leads to a blueshift of the emission. With guided polaritons, there is no energy minimum, and, thus, a faster energy relaxation along the dispersion leads to a redshift of the emission, despite the fact that the dispersion as a whole is blueshifted. This redshift, clearly visible in the movie and in Supplementary Fig. S4 and S5, and well reproduced by simulations based on semi-classical Boltzmann equations (Supplementary Fig. S6), is due to the faster polariton relaxation rates at higher densities.

Interestingly, the lasing mechanism in ZnO-based systems, such as nanowires^42^, has been a matter of debate for quite a long time. Polariton lasing^43^ was considered as a possible mechanism for over a decade^44-46^, but the absence of direct dispersion measurements made it difficult to draw a definite conclusion. The direct dispersion measurements we report enable one to clearly establish the polaritonic origin for the emission above the nonlinear threshold. The temperature dependences between 5 and 300 K for the polariton lasing energy and of the A-exciton energy are shown in Fig. 2g. The difference between the two energies increases slightly, which demonstrates that lasing occurs in more and more photonic polariton states vs. temperature. This behavior is expected from a previous numerical study of this system^32^ and from previous works performed on planar cavities^7,47,26,48^. Indeed, similar to the increase in pumping, the increase of temperature leads to faster relaxation along the polariton branch, which enables lasing to take place at lower polariton states.

Figure 2h shows a comparison of the thresholds measured in W1 (horizontal polariton lasing) and those of a vertical polariton laser measured in a full microcavity displaying a quality factor of 2000 (ref. ^47^), which corresponds to a cavity photon lifetime of 0.4 ps. Thresholds are comparable, and they are even slightly lower for the horizontal polariton laser in a wide temperature range, which can be understood qualitatively as follows. In both cases, the main polariton scattering mechanism was termed “excitonic gain” in the 60 s (ref. ^49^), which involves the scattering of an exciton-like polariton with either another exciton-like polariton or with a LO-phonon towards a polariton state with lower energy and thus with a larger photonic fraction. In vertical cavities, polariton lasing takes place if excitonic gain is efficient enough compared with the polariton lifetime. In a guided mode geometry, the scattering rate should be compared with the transit time of polaritons under the pumping spot. Considering a typical 20 µm size pumping area and polariton guided modes propagating at 13 µm/ps at 5 K and 60 µm/ps at 300 K, the transit time of polaritons under the spot is 1.5 ps and 0.33 ps, respectively (neglecting the feedback provided by the horizontal cavities), comparable with the 0.4 ps of a vertical ZnO-based cavity^47^. This simple estimate explains the similarity of the measured thresholds in the two types of samples, and even the lower threshold of the horizontal polariton laser at 5 K. This estimate also suggests that in thick microcavities, which support both radiative and guided modes, the latter are responsible for strong losses affecting vertical polariton lasing. In a cracked homoepitaxial full cavity (completed with a top dielectric DBR), we have been able to observe lasing simultaneously in the vertical radiative modes and in the horizontal guided modes (not shown).

**Fig. 3:**
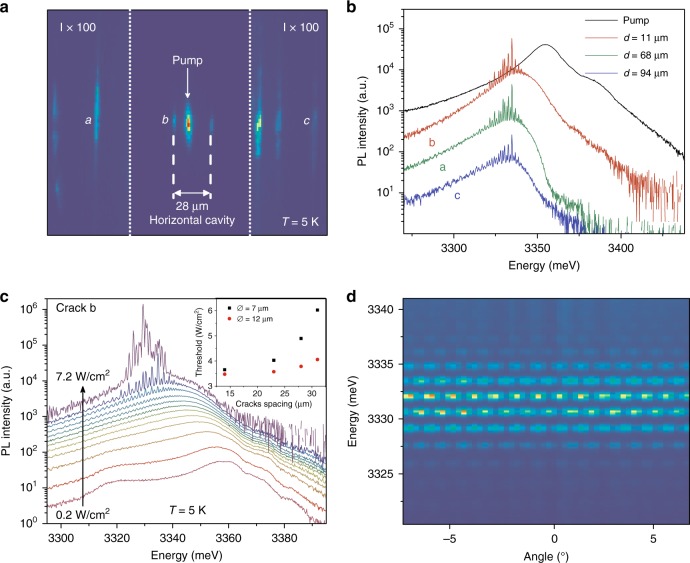
Sample W2. **a** Real space emission at 5K. The intensity (I) of the two lateral regions is magnified by 100. **b** Emission spectra from the pump region and from the cracks labeled “**a**”, “**b**”, and “**c**” in Fig. 3a. *d* is the distance between the crack and the center of the excitation spot. **c** Emission of the crack “**b**” vs. pumping power, showing the emergence of the Fabry-Perot modes of the horizontal cavity together with the onset of lasing (power dependence is shown in Supplemental Material). The inset shows the threshold values vs. the cavity size for two different pump diameters. **d** Far-field image zoomed in onto the fringes resulting from the interference of the emission from two distant cracks, demonstrating their mutual coherence

Another interesting feature is the emergence of sharp emission lines, appearing at threshold within the polariton dispersion. These lines are the Fabry-Perot modes of the horizontal cavity formed by the two cracks surrounding the pump. The rise of these sharp peaks demonstrates the onset of phase coherence for the polariton modes all along the horizontal cavity, as explained below. Figure 3a shows the real space emission at 5K of the sample W2 excited above *P*_th_ by a 7-µm-sized pump (four times smaller than the distance between the surrounding cracks). The emission spectra from the pump area and from the three different cracks are shown in Fig. 3b. The pump area shows the radiative ZnO emission dominated by the first Bragg mode of the mirror at 3355 meV and the D^0^X lines at 3370 meV. The free exciton line is visible as a shoulder at ~3385 meV. The emission from the cracks shows the Fabry-Perot interference peaks. The power-dependent emission spectra measured at crack “b”, demonstrating the emergence of the horizontal Fabry-Perot modes above threshold, are shown in Fig. 3c. The interference fringes are weakly visible in the pump area since there is no well-defined out-coupling mechanism for the guided modes.

Figure 3d is a far-field image (taken from a region without the grating) showing the emission from two distant cracks. The resulting interference pattern shows that at a given energy there is no significant phase fluctuation in the emission from one crack with respect to the other. The mutual coherence extracted from the fringe contrast can reach values close to 70%. The inset of Fig. 3c shows the influence of the spot size and of the distance between cracks on *P*_th_. As one would expect, the smallest threshold is achieved when the pump and cavity size are comparable. However, the dependence is relatively weak, with a pump approximately four times smaller than the cavity showing only a doubled threshold, because the residual absorption in the region without pumping is extremely reduced at the polariton energy. This weak dependence is typical for the polariton lasing mechanism, which enables gain (polariton stimulated scattering) to take place well below the energy of electronic transitions.

The detailed analysis of the Fabry-Perot modes is performed in section IV of supplementary material. The fringe spacing is compatible with the crack spacing, but more than that, the change of the inter-fringe with energy for a known crack separation enables indirect extraction of the dispersion. This method is commonly used in the study of nanowires^44,45^, where direct dispersion measurements are difficult. The result of extraction is shown in Supplementary Fig. S7 and reveals a curved dispersion typical for polaritons with *Ω*_R_~200 meV.

**Fig. 4:**
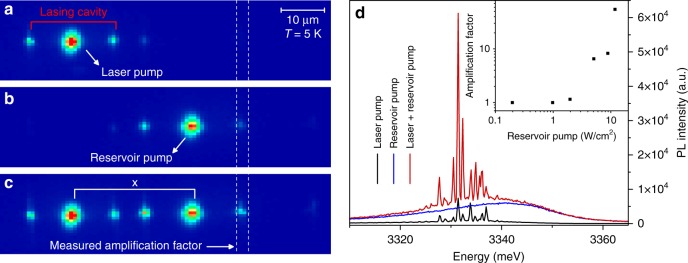
Horizontal polariton amplifier regime at 5K in W2. **a**, **b**, **c** Real space emission with: **a** only “laser pump” (above lasing threshold); **b** only “reservoir pump” (below lasing threshold); **c** both pump spots (“laser” and “reservoir”). The vertical dashed lines show the crack where the amplification is measured. **d** Emission spectra extracted through the crack at the right of the reservoir pump (60 µm from the laser pump). The “reservoir” pump power is 8.9 W/cm^2^, which is below threshold for this specific cavity as one can see from the emission spectrum when only the “reservoir pump” is ON. The “laser” pump power is 6.9 W/cm^2^—above threshold for this cavity. The amplification factor is defined as the ratio between the most intense lasing peaks. The inset shows its evolution as a function of the reservoir pump

Next, we use a two-spot experiment to demonstrate how the horizontal polariton laser emission created in a given cavity can propagate and be re-amplified several tens of microns away by another pump, being itself below the lasing threshold (Fig. 4). A first spot, named “laser pump”, excites a horizontal cavity, creating the laser signal to be amplified (Fig. 4a). A second spot, named “reservoir pump”, is placed in another horizontal cavity, at a distance of 60 µm from the laser pump (Fig. 4b). The power density of the laser pump is fixed above threshold, while the power density of the reservoir pump is kept below threshold. The emission is collected at the crack nearest to the reservoir pump, which shows low emission intensity when only one of the two pumps is ON. Figure 4c shows the configuration when both spots are turned ON. The emission spectra from the selected crack are displayed for each of the three previous pumping schemes in Fig. 4d. The horizontal polariton laser signal is amplified when the two pumps are ON. The amplification factor is calculated as the ratio between the emission maxima measured when both pumps are ON and when only the laser pump is ON. The highest amplification factor is 55 (inset of Fig. 4d), which is reached when the reservoir pump is just below its lasing threshold, demonstrating that the signal created by the laser pump, which can be easily identified by its free-spectral range defined by the initial cavity and which decays by approximately one order of magnitude when reaching the reservoir pump, is strongly re-amplified. This scheme can also be interpreted as an optical transistor, with the transmission of a signal from a source (the laser pump) to a drain (the crack) being modulated by a control gate (the reservoir).

## Discussion

We demonstrated a horizontal polariton laser and amplifier that are much simpler to fabricate and process than full vertical microcavities and are simpler to manipulate than nanowires with which our system shares many similarities. One crucial step for polaritonics is electrical injection^25,50^, which is strongly facilitated in this horizontal geometry. Thus, the whole family of model devices implemented at low temperature in GaAs-based samples can probably be implemented at room temperature under electrical injection using alternative materials (ZnO, GaN, organics, TMD, etc.), while displaying greatly reduced photonic losses due to the guided modes. It should be noted that polariton topological insulators with protected edge states have recently been proposed^18^. Topological polariton lasers have been observed in 1D systems, and topological lasers based on topological in-plane cavities have been realized^39,40^. Topological in-plane arbitrary cavities could probably be implemented for polaritons, which when combined with the present observation could enable realization of 2D topological polariton lasers with a low threshold and high beam quality that can operate at room temperature. These exciting perspectives open up the way to many studies in the future.

## Materials and methods

The samples were studied through micro-photoluminescence using the fourth harmonic (266 nm) of a Nd:YAG laser as the excitation source. The pulse duration was 400 ps and the repetition rate was 20 kHz. To make a comparison with cw excitation, one, therefore, has to multiply the average quasi-cw pumping power we use by a factor of 125,000. A UV microscope objective with a 0.4 numerical aperture was used to obtain small excitation spots with varying diameters. The emission of the sample was collected through the same objective and imaged onto the spectrometer slit by a spherical lens mounted on a motorized translation stage.

## Electronic supplementary material

Supplemental Material

Supplementary material: Video showing photoluminescence versus pumping power

## References

[1] Hopfield JJ (1958). Theory of the contribution of excitons to the complex dielectric constant of crystals. Phys. Rev..

[2] Agranovič VM (1960). Dispersion of electromagnetic waves in crystals. Ž. Èksper Teor. Fiz..

[3] Weisbuch C, Nishioka M, Ishikawa A, Arakawa Y (1992). Observation of the coupled exciton-photon mode splitting in a semiconductor quantum microcavity. Phys. Rev. Lett..

[4] Shelykh IA, Kavokin AV, Rubo YG, Liew TCH, Malpuech G (2010). Polariton polarization-sensitive phenomena in planar semiconductor microcavities. Semicond. Sci. Technol..

[5] Carusotto I, Ciuti C (2013). Quantum fluids of light. Rev. Mod. Phys..

[6] Kasprzak J (2006). Bose-einstein condensation of exciton polaritons. Nature.

[7] Kasprzak J, Solnyshkov DD, André R, Dang LS, Malpuech G (2008). Formation of an exciton polariton condensate: thermodynamic versus kinetic regimes. Phys. Rev. Lett..

[8] Sun YB (2017). Bose-Einstein condensation of long-lifetime polaritons in thermal equilibrium. Phys. Rev. Lett..

[9] Amo A (2009). Superfluidity of polaritons in semiconductor microcavities. Nat. Phys..

[10] Sanvitto D, Kéna-Cohen S (2016). The road towards polaritonic devices. Nat. Mater..

[11] Lagoudakis KG (2008). Quantized vortices in an exciton–polariton condensate. Nat. Phys..

[12] Hivet R (2012). Half-solitons in a polariton quantum fluid behave like magnetic monopoles. Nat. Phys..

[13] Sala VG (2015). Spin-orbit coupling for photons and polaritons in microstructures. Phys. Rev. X.

[14] Lai CW (2007). Coherent zero-state and π-state in an exciton–polariton condensate array. Nature.

[15] Jacqmin T (2014). Direct observation of Dirac cones and a flatband in a honeycomb lattice for polaritons. Phys. Rev. Lett..

[16] Kim NY (2014). *f*-band condensates in exciton-polariton lattice systems. Phys. Rev. B.

[17] Whittaker CE (2018). Exciton polaritons in a two-dimensional lieb lattice with spin-orbit coupling. Phys. Rev. Lett..

[18] Nalitov AV, Solnyshkov DD, Malpuech G (2015). Polariton Z topological insulator. Phys. Rev. Lett..

[19] Solnyshkov DD, Nalitov AV, Malpuech G (2016). Kibble-zurek mechanism in topologically nontrivial zigzag chains of polariton micropillars. Phys. Rev. Lett..

[20] St-Jean P (2017). Lasing in topological edge states of a one-dimensional lattice. Nat. Photonics.

[21] Berloff NG (2017). Realizing the classical XY Hamiltonian in polariton simulators. Nat. Mater..

[22] Imamoğlu A, Ram RJ, Pau S, Yamamoto Y (1996). Nonequilibrium condensates and lasers without inversion: exciton-polariton lasers. Phys. Rev. A..

[23] Christopoulos S (2007). Room-temperature polariton lasing in semiconductor microcavities. Phys. Rev. Lett..

[24] Christmann G, Butté R, Feltin E, Carlin JF, Grandjean N (2008). Room temperature polariton lasing in a GaN-AlGaN multiple quantum well microcavity. Appl. Phys. Lett..

[25] Bhattacharya P (2014). Room temperature electrically injected polariton laser. Phys. Rev. Lett..

[26] Li F (2013). From excitonic to photonic polariton condensate in a ZnO-based microcavity. Phys. Rev. Lett..

[27] Kéna-Cohen S, Forrest SR (2010). Room-temperature polariton lasing in an organic single-crystal microcavity. Nat. Photonics.

[28] Dietrich CP (2016). An exciton-polariton laser based on biologically produced fluorescent protein. Sci. Adv..

[29] Liscidini M, Gerace D, Sanvitto D, Bajoni D (2011). Guided Bloch surface wave polaritons. Appl. Phys. Lett..

[30] Pirotta S (2014). Strong coupling between excitons in organic semiconductors and Bloch surface waves. Appl. Phys. Lett..

[31] Lerario G (2017). High-speed flow of interacting organic polaritons. Light Sci. Appl..

[32] Solnyshkov DD, Terças H, Malpuech G (2014). Optical amplifier based on guided polaritons in GaN and ZnO. Appl. Phys. Lett..

[33] Walker PM (2013). Exciton polaritons in semiconductor waveguides. Appl. Phys. Lett..

[34] Rosenberg I, Mazuz-Harpaz Y, Rapaport R, West K, Pfeiffer L (2016). Electrically controlled mutual interactions of flying waveguide dipolaritons. Phys. Rev. B.

[35] Ellenbogen T, Crozier KB (2011). Exciton-polariton emission from organic semiconductor optical waveguides. Phys. Rev. B.

[36] Ciers J (2017). Propagating polaritons in III-nitride slab waveguides. Phys. Rev. Appl..

[37] Hu F (2017). Imaging exciton-polariton transport in MoSe_2_ waveguides. Nat. Photonics.

[38] Walker PM (2015). Ultra-low-power hybrid lightmatter solitons. Nat. Commun..

[39] Bahari B (2017). Nonreciprocal lasing in topological cavities of arbitrary geometries. Science.

[40] Bandres MA (2018). Topological insulator laser: experiments. Science.

[41] Zuniga-Perez J (2016). Homoepitaxial nonpolar (10-10) ZnO/ZnMgO monolithic microcavities: towards reduced photonic disorder. Appl. Phys. Lett..

[42] Huang MH (2001). Room-temperature ultraviolet nanowire nanolasers. Science.

[43] Zamfirescu M, Kavokin A, Gil B, Malpuech G, Kaliteevski M (2002). ZnO as a material mostly adapted for the realization of room-temperature polariton lasers. Phys. Rev. B.

[44] Chu S, Olmedo M, Yang Z, Kong JY, Liu JL (2008). Electrically pumped ultraviolet ZnO diode lasers on Si. Appl. Phys. Lett..

[45] Vanmaekelbergh D, Van Vugt LK (2011). ZnO nanowire lasers. Nanoscale.

[46] Versteegh MAM, Vanmaekelbergh D, Dijkhuis JI (2012). Room-temperature laser emission of ZnO nanowires explained by many-body theory. Phys. Rev. Lett..

[47] Levrat J (2010). Condensation phase diagram of cavity polaritons in GaN-based microcavities: experiment and theory. Phys. Rev. B.

[48] Jamadi O (2016). Polariton condensation phase diagram in wide-band-gap planar microcavities: GaN versus ZnO. Phys. Rev. B.

[49] Haug H, Grob K (1967). Exciton laser theory. Phys. Lett. A.

[50] Schneider C (2013). An electrically pumped polariton laser. Nature.

