# Supplementary material for: Edge-emitting polariton laser and amplifier based on a ZnO waveguide
Source: Light Sci Appl. 2018 Oct 31;7:82. doi: 10.1038/s41377-018-0084-z (PMC6207564; doi:10.1038/s41377-018-0084-z)
Supplement: Supplementary file 1 — Supplemental Material [file 41377_2018_84_MOESM1_ESM.pdf]

Edge-emitting polariton laser and amplifier based on a ZnO waveguide: Supplementary material

O. Jamadi,[1] F. Réveret,[1] P. Disseix,[1] F. Médard,[1] J. Leymarie,[1] A. Moreau,[1] D. Solnyshkov,[1] C. Deparis,[2] M. Leroux,[2] E. Cambril,[3] S. Bouchoule,[3] J. Zuniga-Perez,[2] and G. Malpuech[1]

[1]PHOTON-N2, Université Clermont Auvergne, CNRS, SIGMA Clermont, Institut Pascal, F-63000 Clermont-Ferrand, France
[2]UCA, CRHEA-CNRS, Valbonne F-06560, France
[3]Centre Nanosciences et Nanotechnologies (C2N), CNRS, University Paris-Saclay, Marcoussis F-91460, France

The supplemental material is divided into four sections. Section I gives more details on the fabrication and the geometry of sample W2 (half-cavity). Section II shows the polariton dispersion below and at threshold, measured with high spatial selection which allows to remove the dispersiveless emission from the cracks. It also shows the raw data allowing to extract the blue shift of polariton modes versus pumping. The emission properties of sample W1 at high pumping densities, and the corresponding numerical simulations based on the solution of semi-classical Boltzmann equations are discussed in Section III. The method used to extract the dispersion for sample W2 and the power dependence of the emission are explained in Section IV.

PACS numbers:

I FABRICATION DETAILS AND SAMPLE W2 GEOMETRY

The sample W1 has been grown by molecular beam epitaxy on m-plane bulk ZnO substrate. It consists of a 1 μm thick $Zn_{1-x}Mg_xO$ (x=0.26) buffer and lower cladding layer, a 50-nm thick ZnO layer, and a 100-nm thick $Zn_{1-x}Mg_xO$ (x=0.26) upper cladding layer (see Fig. 1 of the main text). Thermal mismatch induces cracks as discussed in the main text.

The first-order grating couplers have been fabricated by electron beam lithography using a 80-nm thick, negative-tone Hydrogen silsesquioxane resist. Each grating spans over a 100×100 μm^2 area, and the grating grooves are oriented perpendicularly to the c-axis, that is, parallel to the thermal cracks. The target fill factor is fixed to ~ 50 %, and the periods Λ have been calculated so that the central value of the in-plane wavevector (33 μm^{-1}) is out-coupled perpendicular to the sample surface (Λ =180-220 nm).

The modal confinement factor of the 1D TE_0 guided mode is estimated to be of ~ 46 % in the ZnO guiding core while the overlapping with the grating is estimated to be lower than 1 %. This leads to relatively weak extraction loss, estimated to be lower than 4% of the guided power over a propagation distance of 40 μm (from 2D FDTD simulations).

The sample W2 consists of a bottom DBR comprising 30×(ZnO/ZnMgO) $\lambda/4$ layers (further details on the structural, morphological and optical characterization can be found in [1]), and completed by a 130-nm thick ZnO layer (on top). A cross-section SEM image and the sketch of the sample W2 are shown in Fig. S1(a,b). Both samples display cracks, with a mean distance between them of 25 μm. The surface roughness for both samples was of the order of 1 nm, as obtained from 5×5 μm^2 regions, scanned by atomic force microscopy. The precise values of the layer thicknesses (in the structures) were determined by combining cross- section secondary electron microscopy images and X-ray reflectivity.

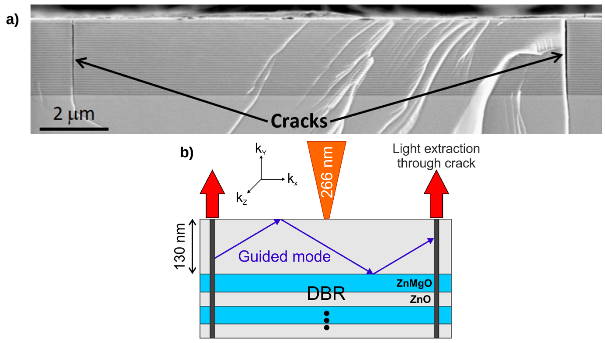

Figure S1: Sample W2. a) SEM image of the half-cavity structure with clearly visible cracks. b) Sketch of a guided mode propagating in the active region.

II SPATIALLY SELECTED EMISSION AND BLUE SHIFT MEASUREMENT

The data shown in Fig. 2 of the main text are taken using a large pumping spot, covering the whole area of a horizontal cavity, which allows to minimize the lasing threshold. It makes difficult spatial selection of emission which therefore contains both light emitted from the grating area, which allows to observe the polariton dispersion, and also light from the cracks, which appears as non-dispersive "horizontal lines" due to diffraction. Fig-

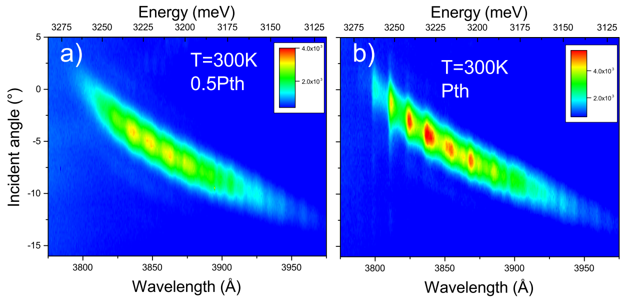

Figure S2: Emission of W1 at 300 K versus energy and emission angle. The emission from the grating is selected by a pinhole which allows to remove the crack emission a) 0.5 P_{th} b) P_{th}

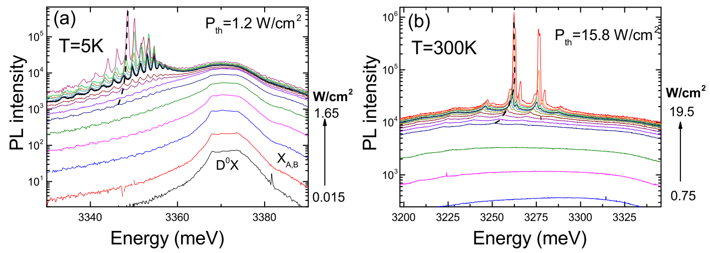

Figure S3: Emission of W1 versus pumping power with spatial selection (grating only): a) 5 K b) 300 K

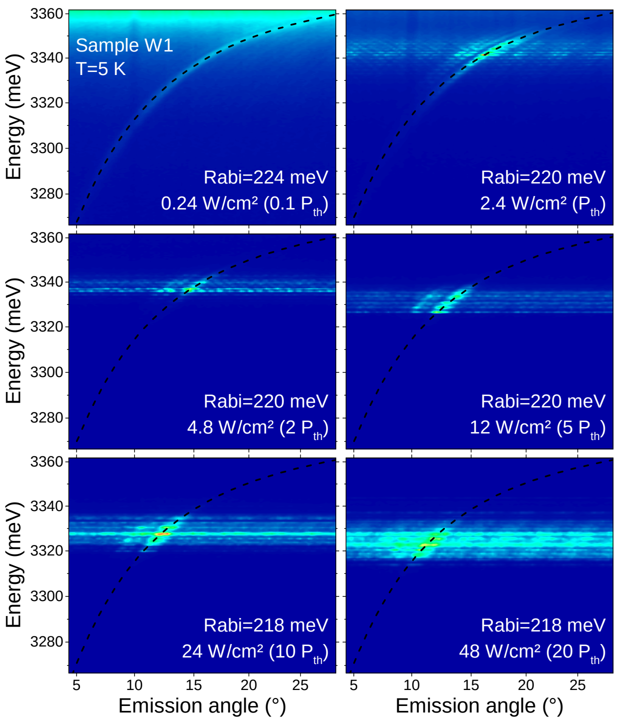

Figure S4: Angularly-resolved PL of W1 at 5 K versus pumping.

ure S2 shows measurements taken at 300 K, both below and at threshold, where a spatial selection of the grating emission by a pinhole is performed. The figure clearly shows the polariton dispersion whereas the dispersionless emission from the cracks has disappeared.

Figure S3 shows the PL spectra of the sample W1 without angular selection, versus pumping power at 5K and 300 K. These figures allows to follow the energy of the Fabry-Perot modes of the horizontal cavity versus pumping, and to track their blue shifts which are shown in Fig. 2(a,b) of the main text.

III HIGH PUMPING DENSITIES

Figure S4 shows the emission of W1, measured in the same conditions as Fig. 2(c,e) of the main text for a pumping power varying from 0.1 to 20 $P_{\rm th}$. One can see that the main emission always fits the polariton dispersion (dashed black curve), even if it becomes harder to determine at the largest pumping densities because of the strong mixing with the dispersionless crack emission. The dispersion itself remains largely unperturbed, showing a slight blue shift of the order of 1-3 meV. The lasing emission above threshold shows a clear red shift by about 20 meV, which corresponds to relaxation towards states with larger and larger photon fractions with increasing pumping power. The same set of data presented slightly differently can be seen in the movie `movie.mp4` (also available at `https://www.youtube.com/watch?v=A0ZSpzKErZw`). In the movie we show both positive and negative angles of emission. It allows to see the two symmetric dispersion curves, corresponding to the positive and negative directions of propagation. Figure S5 is obtained from the same data. It shows the emission spectrum versus energy but without angular resolution taken at different pumping.

It compares qualitatively well with the Figure S6 showing the results of simulations based on the numerical solution of the semi-classical Boltzmann equations for polaritons similar to the ones used in [2]. The general formalism of semi-classical Boltzmann equations and its application to polaritons are discussed in [3].

$$\begin{aligned}\frac{dn_{\mathbf{k}}}{dt} &= P_{\mathbf{k}} - \Gamma_{\mathbf{k}} n_{\mathbf{k}} - n_{\mathbf{k}} \sum_{\mathbf{k}'} W_{\mathbf{k}\to\mathbf{k}'} \left(n_{\mathbf{k}'}+1\right) \quad (1) \\ &+ \left(n_{\mathbf{k}}+1\right) \sum_{\mathbf{k}'} W_{\mathbf{k}'\to\mathbf{k}} n_{\mathbf{k}'}\end{aligned}$$

Here, $n_{\mathbf{k}}$ is the population of polariton or exciton states at a given wavevector, $P_{\mathbf{k}}$ is the pumping (we represent the non-resonant pumping by injection of excitons, assuming that they rapidly thermalize with respect to the free exciton energy with optical phonons), $\Gamma_{\mathbf{k}}$ are the decay rates, determined by the group velocity and the

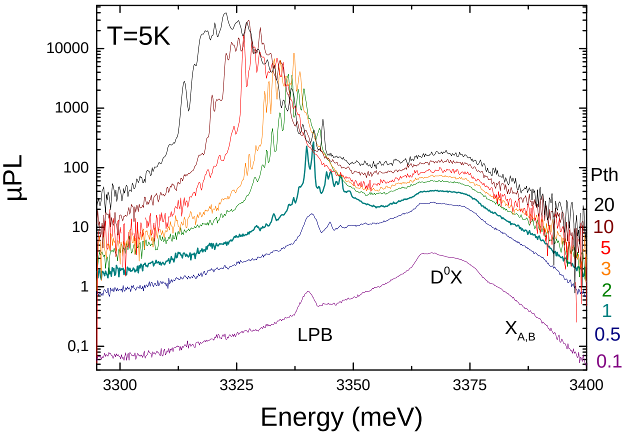

Figure S5: PL of W1 at 5 K versus pumping. The data are the same as the one used for figure S4.

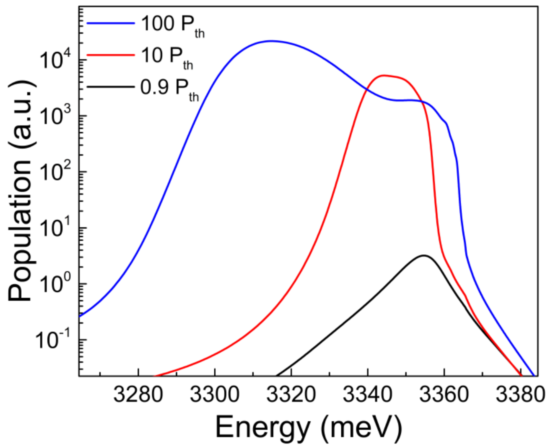

Figure S6: Polariton distribution function (Population) versus energy, calculated as a numerical solution of the semi-classical Boltzmann equation for different pumping powers.

reflection on the cavity edges (we assume 50% reflection probability, as confirmed by COMSOL simulations).

W are the scattering rates between the states, assisted by the exciton-phonon and exciton-exciton interactions. The exciton-phonon scattering rate can be written as:

$$W^{\rm phon}_{\mathbf{k}\to\mathbf{k}'} = \frac{2\pi}{\hbar}\sum_{\mathbf{q}}\left|M\left(\mathbf{q}\right)\right|^2\left(0,1+N^{\rm phon}_{\mathbf{q}}\right) \qquad (2)$$
$$\times\frac{\hbar\gamma_{\mathbf{k}'}/\pi}{\left(E\left(\mathbf{k}'\right)-E\left(\mathbf{k}\right)\pm\hbar\omega_{\mathbf{q}}\right)^2+\left(\hbar\gamma_{\mathbf{k}'}\right)^2}$$

where $M(\mathbf{q})$ is the matrix element of interaction, depending on the phonon type (acoustic or optical), $0,1$ stand for phonon absorption or emission respectively, $N^{\rm phon}_{\mathbf{q}} = 1/(\exp(-E(\mathbf{q})/k_{\rm B}T)-1)$ is the number of phonons with the energy given by the exchanged wavevector $\mathbf{q}$, $\hbar\gamma_{\mathbf{k}'}$ is the broadening of the polariton states (induced by the lifetime or other sources). Thus, at higher temperatures the phonon-assisted processes become enhanced by the increase of the corresponding terms in this scattering rate. On the other hand, the exciton-exciton scattering rate strongly depends on the density of excitons:

$$W^{\rm exc}_{\mathbf{k}\to\mathbf{k}'} = \frac{2\pi}{\hbar}\sum_{\mathbf{q}}\left|M_{\rm exc}\right|^2 N^{\rm exc}_{\mathbf{q}}\left(1+N^{\rm exc}_{\mathbf{q}+\mathbf{k}'-\mathbf{k}}\right)(3)$$
$$\times\frac{\hbar\gamma_{\mathbf{k}'}/\pi}{\left(E\left(\mathbf{k}'\right)-E\left(\mathbf{k}\right)+E\left(\mathbf{q}+\mathbf{k}'-\mathbf{k}\right)-E\left(\mathbf{q}\right)\right)^2+\left(\hbar\gamma_{\mathbf{k}'}\right)^2}$$

We stress that these scattering rates concern only the excitonic fraction of the quasiparticles at any wavevector. Here, $M_{\rm exc}$ is the matrix element of the exciton-exciton scattering, $N^{\rm exc}_{\mathbf{q}}$ is the number of excitons (or exciton-polaritons) with a given wavevector. We see that the overall dependence of the scattering rate on the exciton density is quadratic, which greatly enhances the relaxation at high densities.

The Rabi splitting value is taken as 200 meV. The increase of pumping power enhances all scattering rates for two reasons: 1) the efficiency of exciton-exciton scattering increases with the density of excitons, and 2) bosonic stimulation increases all scattering rates. In planar vertical cavities this enhancement of scattering rate allows to overcome the well-known "bottleneck effect" [4]. In such cavities, the dispersion shows an energy minimum whose depth can be tuned by changing the exciton-photon detuning at zero in-plane wavevector. If the relaxation times are longer than the polariton lifetime near the ground state, polaritons cannot dissipate energy efficiently enough to reach the ground state during their lifetime and they can accumulate higher in energy in the bottleneck region. Increasing pumping power allows to enhance relaxation rates, and to overcome the bottleneck. Eventually, relaxation processes can become efficient enough to lead to the formation of a quasi-thermal distribution function and of a polariton Bose condensate in the dispersion ground state. However, one can engineer the dispersion by changing the exciton-photon detuning. By going to negative detuning, the energy dip in reciprocal space becomes deeper and sharper. It takes longer for polaritons to reach the ground state, and the more photon like polaritons have typically a shorter radiative lifetime. So the pumping density which was good enough to allow condensation in the ground state at positive exciton-photon detuning leads to the formation of a bottleneck at more negative detuning and a further increase of pumping will be needed for polaritons to relax down to the dispersion ground state at this detuning. This picture has been widely discussed theoretically and evidenced experimentally in a large amount of works [5–7] and is well established. In a waveguide geometry, the

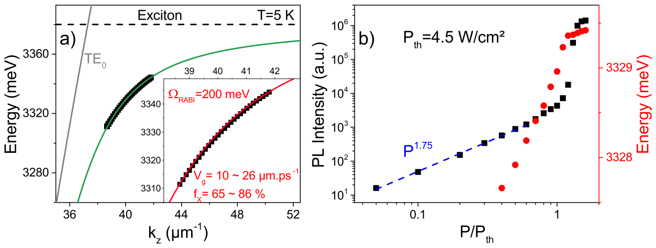

Figure S7: Sample W2. Dispersion of the guided polariton mode and power dependence of emission. a) Experimental dispersion at 5 K extracted from interference patterns (black squares) and the theoretical dispersion calculated using scattering matrix formalism (green). The inset shows the same experimental dispersion fitted with a coupled oscillator model which allows to extract the group velocity and the excitonic fraction of the polariton. b) Emission intensity (black squares) and energy (red dots) of the most intense lasing peak versus pumping power. The exponent of the slope fitting the emission intensity below the pumping threshold (P_{th}) lies between 1 and 2, which indicates a relaxation, assisted both by exciton-exciton and exciton-LO phonon interactions.

picture is quite similar, except that there is no ground state. In this case, the competition between relaxation towards several modes defines the position of the "bottleneck", namely the states which are the most favorable from a loss-gain point of view. When the pumping power is increased, the enhancement of the scattering rates displaces the bottleneck region deeper in energy, leading to a continuous red-shift of the maximum of emission, as observed both experimentally and theoretically.

IV EXTRACTION OF DISPERSION (SAMPLE W2), POWER DEPENDENCE OF EMISSION

The Fabry-Perot modes visible in Fig. 3 of the main text result from the horizontal confinement of light between the two cracks surrounding the excitation spot. These two cracks form an horizontal cavity. The energy spacing between the peaks is determined by the length of the cavity and the slope of the dispersion relation. By knowing the length of the cavity L, we can calculate the difference $\delta k_z = \pi/L$ between subsequent modes. Knowing the energy and the wavevector spacing between modes, one simply needs a reference wavevector k_z to extract the experimental dispersion. It is approximately estimated by adjusting the experimental points to the theoretical dispersion. The result of this procedure is shown in Fig. S7(a).

The theoretical dispersion of the guided modes of both samples was calculated using the scattering matrix formalism [8] taking into account all the sample layers. We choose the bare photonic mode TE_0 by default. The refractive index of the ZnMgO alloy was measured by ellipsometry. The validity of the thickness and the refractive index were confirmed by reflectivity experiments and transfer matrix calculation. The exciton oscillator strength for ZnO has been chosen in agreement with [9].

Another way to calculate the dispersion curve is based on the coupled oscillator model. It also provides a good agreement with experimental results and allows to determine the exciton and photon fractions of the amplified modes. The corresponding curves are shown in Fig. 2 of the main text for the sample W1 and in Fig. S7(a) for the sample W2 (red curve on the inset).

The power dependence of emission extracted from the data presented in Fig. 3(a) of the main text is shown in Fig. S7(b), together with the energy shift of the Fabry-Perot modes which is of the order of 1.5 meV at 2 P_{th} (less than 1% of the Rabi splitting Ω).

[1] J. Zuniga-Perez, L. Kappei, C. Deparis, F. Reveret, M. Grundmann, E. de Prado, O. Jamadi, J. Leymarie, S. Chenot, and M. Leroux, Appl. Phys. Lett. **108**, 251904 (2016).

[2] D. D. Solnyshkov, H. Tercas, and G. Malpuech, Appl. Phys. Lett. **105**, 231102 (2014).

[3] A. Kavokin and G. Malpuech, *Cavity polaritons* (Elsevier, 2003), ISBN 978-0-12-533032-9.

[4] F. Tassone, C. Piermarocchi, V. Savona, A. Quattropani, and P. Schwendimann, Phys. Rev. B **56**, 7554 (1997), URL `https://link.aps.org/doi/10.1103/PhysRevB.56.7554`.

[5] J. Kasprzak, D. D. Solnyshkov, R. André, L. S. Dang, and G. Malpuech, Phys. Rev. Lett. **101**, 146404 (2008), URL `https://link.aps.org/doi/10.1103/PhysRevLett.101.146404`.

[6] J. Levrat, R. Butté, E. Feltin, J.-F. m. c. Carlin, N. Grandjean, D. Solnyshkov, and G. Malpuech, Phys. Rev. B **81**, 125305 (2010), URL `https://link.aps.org/doi/10.1103/PhysRevB.81.125305`.

[7] F. Li, L. Orosz, O. Kamoun, S. Bouchoule, C. Brimont, P. Disseix, T. Guillet, X. Lafosse, M. Leroux, J. Leymarie, et al., Phys. Rev. Lett. **110**, 196406 (2013), URL `https://link.aps.org/doi/10.1103/PhysRevLett.110.196406`.

[8] J. Defrance, C. Lemaitre, R. Ajib, J. Benedicto, E. Mallet, R. Polles, J.-P. Plumey, M. Mihailovic, E. Centeno, C. Ciraci, et al., Journal of Open Research Software **4**, 13 (2016).

[9] O. Jamadi, F. Réveret, E. Mallet, P. Disseix, F. Médard, M. Mihailovic, D. Solnyshkov, G. Malpuech, J. Leymarie, X. Lafosse, et al., Phys. Rev. B **93**, 115205 (2016), URL `https://link.aps.org/doi/10.1103/PhysRevB.93.115205`.
